# Risk Factors for Chemotherapy-Induced Peripheral Neuropathy Caused by Nanoparticle Albumin-Bound Paclitaxel in Advanced Breast Cancer

**DOI:** 10.1155/2022/9430952

**Published:** 2022-09-13

**Authors:** Qie Guo, Haonan Zhang, Xiao Li, Xianghua Quan

**Affiliations:** ^1^Department of Clinical Pharmacy, The Affiliated Hospital of Qingdao University, China; ^2^Zibo City Zhangdian District People's Hospital, China

## Abstract

Breast cancer (BC) is one of the most common malignancies affecting women and the leading cause of related mortality worldwide. An estimated 2260000 new cases of BC were diagnosed in 2020, which have seriously threatened the health. Paclitaxel (PTX), a natural product isolated from the bark of the pacific yew, has been found to be effective in treating advanced BC. Chemotherapy-induced peripheral neuropathy (CIPN), which refers to the damage to the peripheral nerves caused by exposure to a neurotoxic chemotherapeutic agent, is a common side effect affecting the patients undergoing PTX chemotherapy. Significant research efforts are needed to identify the various risk factors associated with CIPN. Here, a univariate analysis in BC patients with nanonab-PTX treatment was performed. The rate of CIPN in BC patients with albumin-bound paclitaxel (nab-PTX) for more than four weeks was significantly higher than that of patients with chemotherapy for less than four weeks. Moreover, the rate of CIPN in BC patients receiving nab-PTX first-line chemotherapy was remarkably higher than that in BC patients receiving paclitaxel as a sequence scheme. Taken together, chemotherapy cycles and the priority of nab-PTX-based chemotherapy can be considered the potential risk factors for CIPN induced by nab-PTX.

## 1. Introduction

Breast cancer (BC) is the most common cancer affecting women [1]. BC survival rates have increased, and the number of deaths associated with this disease has been steadily declining, primarily due to different factors such as earlier detection and a better understanding of the disease [2]. BC is an extremely heterogeneous disease composed of multiple unique histologic subtypes that harbor distinct molecular signatures [3]. BC has been classified into distinct histological groups, with the two most common subtypes being invasive ductal carcinoma (IDC) and invasive lobular carcinoma (ILC) [4]. The continuous growth of the clonal subpopulations can effectively guide the cell differentiation into four varieties, including Luminal A (ER and/or PR positive and HER2 negative), Luminal B (ER and/or PR positive and HER2 positive), HER2-enriched (ER and PR negative, and HER2 positive), and Basal-Like (triple negative breast cancer ER, PR, and HER2 negative), in accordance with the expression of estrogen receptor (ER), progesterone receptor (PR), and overexpression of human epidermal growth factor receptor 2 (HER2/neu) [5, 6]. Advance in BC therapy has been identified. Ductal carcinoma in situ can progress to invasive cancer and is treated with breast-conserving surgery and radiation therapy without further lymph node exploration or systemic chemotherapy. Node-positive breast cancer is treated systemically with chemotherapy, endocrine therapy (for hormone receptor-positive cancer), and trastuzumab (for cancer overexpressing ERBB2) [7]. Although the surgical removal remains the curative treatment, systemic chemotherapy is an optimal therapeutic strategy for BC patients who are diagnosed at an advanced stage and consequently display distant metastases and poor prognosis, thus offering better outcomes than surgery alone. Moreover, chemotherapy is used before or after surgery in early stage breast cancer and surgery cannot cure metastatic disease [8]. Paclitaxel (PTX) and its derivative preparations nanoparticle albumin-bound paclitaxel (nab-PTX) have been commonly employed in the chemotherapy for the metastatic BC with good survival benefits [9]. Chemotherapy-induced peripheral neuropathy (CIPN) is a well-known nonhematological adverse effect of PTX, which can adversely affect optimal treatment of active disease, thereby leading to dose reduction and even premature cessation of thus chemotherapy and inducing long-term debilitating effects with increased morbidity as well as decreased quality of life [10]. In addition, one of the most frequent dose-limiting complications arising from PTX treatment is the emergence of CIPN, and the clinical management of this condition has been proved to be difficult [11]. Thus, exploration of the various risk factors that can promote CIPN development can be significant to furnish an effective strategy for the prevention of CIPN. Herein, the clinicopathological features including age, body mass index (BMI), body surface area (BSA), pathological type, clonal subpopulations, Eastern Cooperative Oncology Group (ECOG) score, usage and dosage, chemotherapy cycles, chemotherapy regimens, metastatic lesions, and the previous diseases in patients with advanced BC were identified, and the severity of CIPN was retrospectively analyzed. Chemotherapy cycles and the priority of nab-PTX-based chemotherapy were identified as putative risk factors for CIPN, which can be used in the clinical practice for preventing CIPN with prolongation of the quality of life of the patient on a long-term basis.

## 2. Materials and Methods

### 2.1. Subjects

Ninety patients with advanced BC ranging in age from 18 to 80 years were enrolled. All of the patients were treated at the Affiliated Hospital of Qingdao University between January 2018 and December 2020 and were characterized as having the injection of nab-PTX. The various clinicopathological features of BC patients are shown in Table 1. The patients were taken into account based on the inclusion criteria as follows: ECOG score ≤ 2 and survival time > 3. The detection of the blood routine, renal function, and liver function in the patients was performed to ensure that chemotherapy was carried out on schedule. The patients with incomplete case data, or with neuropathy before starting treatment using PTX and nab-PTX, were excluded from the present study. Alternatively, the patients allergic to any PTX or nab-PTX were not recruited in this study.

This study was approved by the ethics committee of the Affiliated Hospital of Qingdao University. Informed written consent was obtained from all patients.

### 2.2. Follow-Up Visits to Assess the Various Complications in Patients

Follow-up visits were executed, and the following observations were carried out:

1. The patient's specific medication plan, medication time, and the cumulative dose of drugs
2. Types, severity, and occurrence time of adverse reactions during the chemotherapy
3. Determination of the baseline characteristics such as age, BMI, BSA, ECOG score, pathological type, clonal subpopulations, chemotherapy cycles, chemotherapy regimens, number of metastatic parts, and the previous diseases

### 2.3. The Assessment of Adverse Reaction in BC Patients

Adverse reactions and neurotoxicity of BC patients before, during, and after the chemotherapy were evaluated based on the Common Terminology Criteria for Adverse Events (National Cancer Institute (NCI)) version 5.0 (CTCAE V5.0). The severity of CIPN in BC patients was analyzed by the same neurologist and determined using the following grading method:

- Grade I: asymptomatic and disappearance of tendon reflex or abnormal sensation (tingling sensation)
- Grade II: sensory abnormalities (tingling sensation) and limited limb function
- Grade III: sensory abnormalities (tingling sensation) and frustrations in the daily living
- Grade IV: forensic function loss
- Grade V: death

### 2.4. Statistical Analysis

Statistical analysis was performed using SPSS 22.0. Counting data analysis was performed using *χ*^2^ tests. ^∗^*p* < 0.05 was considered statistically significant.

## 3. Results

### 3.1. Analysis of Adverse Conditions of Patients

The adverse reactions of patients using nab-PTX mainly involve the blood system, nervous system, digestive system, and neuromuscular and respiratory systems. The adverse reactions of patients are mostly grades 1-2, and the overall resistance has been found to be good. The incidence rate of toxicity and neurotoxicity was higher, 77 cases (85.6%) had neutropenia, 38 cases (42.2%) had leukopenia, 75 cases (83.3%) had thrombocytopenia, and 83 cases (92.2%) had anemia, whereas 53 cases had CIPN (58.8%) (Supplementary Table 1).

### 3.2. Severity Level and Occurrence Time of CIPN

**Figure 1:**
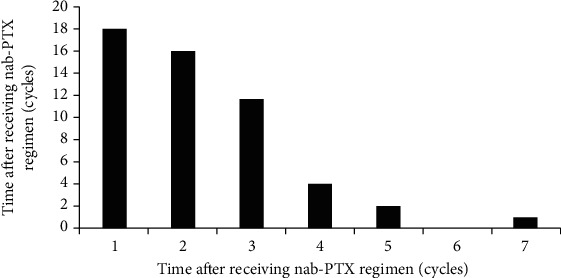
The occurrence of CIPN after receiving nab-PTX regimen (cycles).

There were 10 cases of CIPN of grade I, 30 cases of grade II, and 13 cases of grade III, but no patients displayed CIPN of grade IV (Table 2). CIPN occurred in 18 cases after the first cycle of chemotherapy, 16 cases after the second cycle of chemotherapy, and 12 cases after the third cycle of chemotherapy (Figure 1). Overall, the patients mainly developed CIPN during 1-3 cycles since the chemotherapy initiation, thus accounting for 64% of the total number of CIPN.

### 3.3. Univariate Analysis of the Various Risk Factors Associated with CIPN

The patients were divided into the neurotoxicity group (53 cases) and the nonneurotoxicity group (37 cases) according to whether the neurotoxicity occurred. Age, BMI, BSA, pathological type, clonal subpopulations, ECOG score, usage and dosage, chemotherapy cycles, chemotherapy regimens, metastatic lesions, and the previous diseases were considered variables. The *χ*^2^ test was performed on the baseline data of the two different groups of patients, and the comparison of data between the CIPN group and the nonneurotoxicity group is shown in Table 3. In patients receiving the first-line nab-PTX, the rates of CIPN were 37.7% vs. 62.3% in patients receiving later rounds of treatment (*p* = 0.042). Furthermore, the rates of CIPN in the patients after completing 1 (or 2 or 3) cycles of nab-PTX-based chemotherapy were 32% vs. 68% in patients receiving CIPN for more than four cycles (*p* = 0.037). But there were no significant differences between the remaining groups (*p* > 0.05) (Table 3). The results demonstrated that the chemotherapy cycles and the priority of nab-PTX-based chemotherapy acted as potential risk factors of the incidence of CIPN.

## 4. Discussion and Conclusion

It has been found that compared with the solvent PTX, nab-PTX can exhibit better antitumor effect and lower tendency to develop resistance. Thus, it can significantly improve the survival rate of patients and has become a key drug for the treatment of advanced breast cancer [12, 13]. PTX primarily acts by disrupting the cell division mediated by microtubules which function as tracks for axonal transport, and PTX can effectively interrupt this process, thereby resulting in CIPN [14]. CIPN is usually initiated with paresthesia, which is a lesser degree of pain in the extremities [15]. It can later develop into loss of sensory perception, motor impairment, and autonomic nervous dysfunction, with patients often presenting the sensory symptoms of a “sock and glove” distribution in the upper and lower limbs that can then spread proximal to the body [16]. Atypical symptoms in patients with CIPN include dull sensation, numbness, burning, shooting or electric shock sensation, hyperalgesia, and ectopic pain which can be induced by mechanical and/or thermal stimulation [17]. Such manifestations as perioral numbness, autonomic neuropathy, paclitaxel-related acute pain syndrome, seizures, transient encephalopathy, and phantom limb pain have been reported to be rare but cannot be completely ignored [18]. Additionally, CIPN can adversely affect the quality of life of patients to varying degrees and even lead to paralysis and disability [19]. Indeed, 70% of patients receiving nab-PTX chemotherapy will experience CIPN, and 10% of patients will experience CIPN of grade 3 or higher [20, 21]. CIPN might appear at the first round of chemotherapy treatment, and the symptoms usually improve after chemotherapy is stopped, or it may last for more than 1 year [22]. Previous studies have confirmed that the solvent polyoxyethylene castor oil of solvent PTX can cause axonal swelling, degeneration, and demyelination and then cause persistent neuropathy [23]. Polysorbate-80 can also cause the degeneration of neuronal vesicles, thereby inducing PTX-mediated neurotoxicity [24]. Albumin-bound paclitaxel with good curative effect and reduced toxicity characteristics has been successfully developed since the potential use of nanoparticle agents in cancer treatment enjoys an high profile [25, 26]. Consistently, the water solubility and treatment index have been highly improved, accompanied by the reduction of neuropathy caused by solvents [27, 28]. However, there are currently different opinions on the various risk factors associated with the incidence of CIPN.

A phase II clinical trial of advanced non-small-cell lung cancer confirmed that advanced age is a major risk factor for CIPN [29]. Older patients had a significantly higher risk of dose adjustment on PTX due to CIPN [30]. However, there was no significant difference in the incidence of CIPN in the ≥55 group compared with that in the <55 group for each additional year of age. In fact, aging caused increased expression of cytoskeletal proteins and decreased axonal transport in peripheral nerves, thereby resulting in decreased sensory discrimination and muscle strength [31, 32]. Here, no statistical association between the age and the rates of CIPN occurrence was observed, but aging may be an intrinsic factor in the incidence of CIPN.

High BMI is also considered to be one of the important factors of PTX-induced CIPN. [33, 34] Here, the univariate analysis suggested that BMI had no significant correlation with CIPN. BSA also served as a putative risk factor for predicting the neuropathy in patients with PTX- and oxaliplatin-based chemotherapy [32, 35, 36]. However, our findings showed that there was no association between BSA and the incidence of CIPN using the univariate analysis. Uniform conclusions were not maintained in the final model; thus, it can be concluded that the effect of BMI and BSA on CIPN is controversial. Notably, the higher chemotherapy dose should be administrated in the patients with higher BSA, indicating that BSA may not be an independent risk factor for CIPN occurrence. Additionally, the BSA and BMI are correlated though the association was not perfectly, thus suggesting that more samples are needed for further validation.

A number of studies have reported that the incidence rate of CIPN was not statistically significant with the 3-week dosing regimen compared to the weekly regimen [37, 38]. However, once a week dose of PTX can lead to the higher neurotoxicity. A meta-analysis confirmed that there were no significant differences in the effect of different doses of nab-PTX on the peripheral neuropathies, but the incidence rate of severe CIPN induced by Q3W dosing regimen was markedly lower than that caused by QW dosing regimen [27]. Nevertheless, a retrospective study demonstrated the opposite results that Q3W dosing regimen resulted in more severe CIPN compared to QW dosing regimen [39]. In this study, no correlation was also found between the dosing regimen and the incidence of CIPN. Further studies should be performed to determine the effect of dosing regimen on the incidence of CIPN using more analytical methods with comprehensive inclusion of multiple variables.

The cumulative dose and chemotherapy regimen have also been related to the incidence of CIPN [40]. When the cumulative dose of PTX is greater than 1500 mg/m^2^, it can significantly increase the risk of CIPN [41]. Herein, it is important to note that the incidence of CIPN in the patients with ≥4 weekly chemotherapy is obviously higher compared with that in the patients treated with nab-PTX for less than 4 weeks. Consistently, it can be inferred that the cumulative accumulation of nab-PTX over the course of prolonged chemotherapeutic treatment is likely to have contributed to the emergence of CIPN.

As recognized, paclitaxel acts as a microtubule stabilizer to disrupt mitosis and thus plays an anticancer role [42]. The changes in microtubule dynamics operates impaired neuronal transport of organelles, nutrients, and neurotransmitters through axons. Paclitaxel also advanced the injury of middle dorsal root ganglion (DRG) [43] and the activation of microglial cells in the spinal cord [44]. In addition, paclitaxel can initiate the “reverse death” process of long-distance transfer of cellular components between neuron cell body and nerve endings by microtubules [45]. Here, our findings showed that the rates of CIPN in patients receiving the first-line nab-PTX were distinctly higher than those in patients receiving later rounds of treatment. To the best of our knowledge, there is the first published evidence suggesting that the priority of nab-PTX-based chemotherapy can serve as a putative risk factor of the incidence of CIPN, thus offering a potentially new insight into the risk profile associated with nab-PTX-based chemotherapy. It is reasonable to infer that the early capacity using of nab-PTX sensitizes the DRG, which advances the disability of microtubule function and triggers CIPN.

The chemotherapy regimen of the breast cancer patients is often combined with cisplatin, and platinum compounds are likely to cause substantial neurotoxicity [46]. Almost all patients appeared to develop neuropathy when cisplatin cumulative dose reached 500-600 mg/m^2^ [47, 48]. However, no correlation of chemotherapy regimen and period with the incidence of CIPN was observed, which indicated that the retrospective investigation and logistic regression analysis should be employed.

However, many limitations in this study should not be ignored. Firstly, the number of samples was relatively small, and the research object was confined to a single diagnosis and treatment center. The research results may be biased due to regional restrictions. Secondly, only patients with advanced breast cancer were included in this study. Due to the particularity of group of patients analyzed, there may be defects such as incomplete information collection and relatively small sample size. Furthermore, the chemotherapy cycle of some patients is short, which fails to meet the chemotherapy cycle length of advanced patients, and the lack of follow-up may reduce the incidence and severity of neurotoxicity. Additionally, the determination is based on the doctor's assessment and the patient's report; thus, the frequency and severity of neurotoxicity may be underestimated. The subsequent studies will further expand the sample size and continue to track the observations which can be judged through objective and sensitive methods or instruments. In summary, identifying the pivotal risk factors can facilitate the timely adjustment of chemotherapy decisions, minimize the occurrence of CIPN, and markedly reduce the output of medical expenses related to the incidence of CIPN. Moreover, the occurrence of CIPN should be closely monitored for high-risk patients with incidence of CIPN, to provide an optimal reference for the relevant personalized clinical treatment.

## Figures and Tables

**Table 1:** Clinicopathological features of BC patients.

Clinical features	Number	Percentage (%)
Age		
<55	52	57.7
≥55	38	42.2
BMI		
<24.9	56	62.2
≥25	34	37.7
BSA		
≤1.6	13	14.4
>1.6	77	85.5
Pathological type		
IDC	57	63.3
Others	33	36.6
Clonal subpopulations		
HER2+	10	11.1
TNBC	34	37.8
Luminal A	6	6.7
Luminal B (HER2-)	34	37.8
Luminal B (HER2+)	6	6.7
ECOG score		
0	9	10
1	68	74.4
2	14	15.5
Usage and dosage		
200 mg QW	62	68.8
400 mg Q3W	28	31.1
Chemotherapy cycles		
≤4 cycles	37	41.1
>4 cycles	53	58.8
Chemotherapy regimens		
Single	36	40
Combination	54	60
Metastatic lesions		
1~2	44	48.8
≥3	46	51.1
Previous diseases		
Diabetes	9	10
Hypertension	15	16.6
Others	12	13.3

BMI: body mass index; BSA: body surface area; ECOG: Eastern Cooperative Oncology Group.

**Table 2:** The classification for BC patients with nab-PTX chemotherapy.

Total numbers	Patients without CIPN	Patients with CIPN		
90	37	53		
		Grade I	Grade II	Grade III
		10	30	13

**Table 3:** A univariate analysis performed on the CIPN group and non-neurotoxicity group (*n* = 90).

Group	CIPN patients (53)	Non-neurotoxicity patients (37)	*p* value
Age			0.143
<55	34 (64.1)	18 (48.6)	
≥55	19 (35.8)	19 (51.3)	
BMI			0.188
<24.9	30 (56.6)	26 (70.2)	
≥25	23 (43.3)	11 (29.7)	
BSA			0.106
<1.6	5 (9.4)	8 (21.6)	
≥1.6	48 (90.5)	29 (78.3)	
Pathological type			0.127
IDC	37 (69.8)	20 (54)	
Others	16 (30.1)	17 (45.9)	
Clonal subpopulations			0.09
HER2+	7 (13.2)	3 (8.1)	
TNBC	22 (41.5)	12 (32.4)	
Luminal A	5 (9.4)	1 (2.7)	
Luminal B (HER2-)	14 (26.4)	20 (54)	
Luminal B (HER2+)	5 (9.4)	1 (2.7)	
ECOG score			0.299
0~1	43 (81.3)	33 (89.1)	
2	10 (18.8)	4 (10.8)	
Usage and dosage			0.821
200 mg QW	37 (68.5)	25 (67.5)	
400 mg Q3W	16 (29.6)	12 (32.4)	
Chemotherapy cycles			0.037
≤4 cycles	17 (32)	20 (54)	
>4 cycles	36 (68)	17 (45.9)	
Chemotherapy regimens			0.336
Single	19 (35.8)	17 (45.9)	
Combination	34 (64.1)	20 (54)	
First-line treatment of nab-paclitaxel			0.042
Yes	20 (37.7)	22 (59.5)	
No	33 (62.3)	15 (40.5)	
Metastatic lesions			0.641
1~2	27 (50.9)	17 (45.9)	
≥3	26 (49)	20 (54)	
Previous diseases			0.431
Yes	23 (43.4)	13 (35.1)	
No	30 (56.6)	24 (64.9)	

BMI: body mass index; BSA: body surface area; ECOG: Eastern Cooperative Oncology Group.

## Data Availability

The data used to support the findings of this study are available from the corresponding author upon request.

## Supplementary Materials

Supplementary Materials Supplementary Table 1: summary of adverse reactions in BC patients with nab-PTX chemotherapy.
