# Supplementary material for: Risk Factors for Chemotherapy-Induced Peripheral Neuropathy Caused by Nanoparticle Albumin-Bound Paclitaxel in Advanced Breast Cancer
Source: Biomed Res Int. 2022 Sep 13;2022:9430952. doi: 10.1155/2022/9430952 (PMC9489370; doi:10.1155/2022/9430952)
Supplement: Supplementary Materials — Supplementary Table 1: summary of adverse reactions in BC patients with nab-PTX chemotherapy. [file 9430952.f1.doc]

**Supplementary Table 1 Summary of adverse reactions in BC patients with nab-PTX chemotherapy**

| Types of adverse reactions |  | Total  Number(%) | Number(%) of Grade Ⅲ and Grade Ⅳreactions |
| --- | --- | --- | --- |
| Nervous system |  |  |  |
|  | Neurotoxicity | 53（58.8％） | 13（14.4％） |
|  | Dizzy | 5（5.6％） | 0 |
|  | Headache | 4（4.44％） | 0 |
| Skin |  |  |  |
|  | Erythra | 9（10％） | 0 |
|  | Nail blackening | 5（5.6％） | 0 |
| Neuromuscular and skeletal |  |  |  |
|  | Physical fatigue | 27（30％） | 0 |
|  | Myalgia | 18（20％） | 4（4.4％） |
|  | Arthralgia | 16（17.8％） | 2（2.2％） |
| Cardiovascular system |  |  |  |
|  | Peripheral edema | 12（13.3％） | 2（2.2％） |
|  | Palpitation | 3（3.3％） | 0 |
| Gastrointestinal system |  |  |  |
|  | Constipation | 14（15.5％） | 1（1.1％） |
|  | Nausea | 11（12.2％） | 0 |
|  | Vomiting | 9（10％） | 0 |
|  | Abdominal distension | 8（8.9％） | 0 |
|  | Diarrhea | 7（7.8％） | 0 |
|  | Abdominal pain | 5（5.6％） | 0 |
| Eye |  |  |  |
|  | Dryness | 2（2.2％） | 0 |
|  | Conjunctivitis | 2（2.2％） | 0 |
| Respiratory system |  |  |  |
|  | Cough | 13（14.4％） | 0 |
|  | Expectoration | 5（5.6％） | 0 |
| Hematotoxicity |  |  |  |
|  | Neutropenia | 77（85.6％） | 16（17.8％） |
|  | Thrombocytopenia | 38（42.2％） | 8（8.9％） |
|  | Leukocytopenia | 75（83.3％） | 13（14.4％） |
|  | Anemia | 83（92.2％） | 11（12.2％） |
| Others |  |  |  |
|  | Fever | 10（11.1％） | 0 |
